# The characteristics of patients with mycobacterium tuberculosis blood stream infections in Beijing, China: a retrospective study

**DOI:** 10.1186/s12879-016-2084-z

**Published:** 2016-12-12

**Authors:** Xiaoqing Liu, Sainan Bian, Yueqiu Zhang, Lifan Zhang, Qiwen Yang, Peng Wang, Yingchun Xu, Xiaochun Shi, Yao Zhang, Roy F. Chemaly

**Affiliations:** 1 Department of Infectious Diseases, Peking Union Medical College Hospital, Peking Union Medical College, Chinese Academy of Medical Sciences, No.1 Shuaifuyuan, Dongcheng District, Beijing, 100730 China; 2 Clinical Epidemiology Unit, International Epidemiology Network, Peking Union Medical College, No.1 Shuaifuyuan, Dongcheng District, Beijing, 100730 China; 3 Laboratory Department, Peking Union Medical College Hospital, Peking Union Medical College, Chinese Academy of Medical Sciences, No.1 Shuaifuyuan, Dongcheng District, Beijing, 100730 China; 4 Department of Infectious Diseases, Infection Control, and Employee Health, Unit 1460, The University of Texas MD Anderson Cancer Center, 1515 Holcombe Blvd, Houston, TX 77030 USA

**Keywords:** Mycobacterium tuberculosis, Bacteremia, Blood stream infection

## Abstract

**Background:**

Published information regarding the clinical characteristics, laboratory findings, and outcomes of patients with Mycobacterium tuberculosis (MTB) blood stream infection (BSI) is limited. We aimed in this study to evaluate the clinical characteristics, laboratory evaluation, and outcomes of patients with MTB BSI.

**Methods:**

All patients diagnosed with MTB BSI at Peking Union Medical College Hospital between January 2008 and May 2014 were identified by examining the electronic database listing results of all blood cultures. Data on demographics, clinical characteristics, laboratory manifestations, management, and outcomes were abstracted from medical records.

**Results:**

Six thousand nine hundred seventy-four patients had mycobacterial blood cultures during the study period. Of 48 patients (0.7%) with MTB BSI, 26 patients (54%) were considered to be immunocompromised (refers to a person who has a significantly impaired immune system). This was due to human immunodeficiency virus (HIV) infection (*n* = 2 of 48 tested), receiving steroids (*n* = 17, including 16 with rheumatic diseases and one with myasthenia gravis), malignancy (*n* = 3), diabetes mellitus (*n* = 3), and renal transplantation (*n* = 1). The main clinical manifestations were fever (100%, with a median of 40 °C), weight loss (48%) and cough with sputum production (46%). Most patients had one or more organs involved (81%). The median time from onset of fever to diagnosis was 8 weeks (IQR 5 ~ 14). Six patients died within 1 week after diagnosis. Of the 17 patients completing treatment, 14 patients (82%) recovered without major complications and they had a shorter time interval between onsets of symptoms to treatment compared to those died of TB.

**Conclusions:**

In this group of patients with MTB BSI, fever and multiple organs involvement were common, the outcome was poor and timely diagnosis and treatment might favor outcome.

## Background

Developing countries like China face a challenging combination of an expanding epidemic of human immunodeficiency virus (HIV) infection, cancer, and persistently high prevalence of tuberculosis (TB) [1]. These combinations may place a growing proportion of its population at risk for severe forms of Mycobacterium tuberculosis (MTB) infections, including dissemination and MTB blood stream infection (BSI) (refers to those with positive MTB blood cultures) with subsequent poor outcomes.

Hematogenous, disseminated, or miliary tuberculosis has been used exchangeably in the literature describing in most instances a lymphohematogenous spread of MTB. Miliary tuberculosis accounts for about 1–2% of all cases and about 8% of all forms of extrapulmonary TB in immunocompetent individuals [1].

Reports on MTB BSI are scarce with most published data emerging from the HIV infected patients [2, 3]. Few studies report MTB BSI among a general population [4, 5]. With the use of liquid culture methods in recent years, it became possible to isolate MTB from blood culture bottles. In a prospective cohort of 368 HIV-infected patients hospitalized with severe sepsis [3], 86 patients (23%) had MTB BSI, and the 30-day mortality of patients with or without MTB BSI was 53.2 and 30.7%, respectively. In a study enrolling 238 consecutive febrile adult patients, 173 (73%) were HIV-positive and 67 (28%) had BSI, showing that MTB ranked second at 19% as etiology of bloodstream infections [4]. In another study of 57 patients (55 were HIV positive) with MTB BSI, 42% died within 1 month of hospitalization [5].

Because of the high mortality associated with MTB BSI, we aimed in this study to better understand the clinical and laboratory manifestations and the impact of this infection among patients who were admitted to our institution, a major national referral hospital in Beijing, China.

## Methods

### Study population

All patients diagnosed with MTB BSI at Peking Union Medical College Hospital between January 2008 and May 2014 were identified by examining the electronic database listing results of all blood cultures. Data on demographics, clinical characteristics, underlying diseases, laboratory manifestations, management, and outcomes were abstracted from medical records.

Criteria for determining who gets mycobacterial blood culture was fever last for more than 3 weeks, temperature above 38.3 °C, and still not being diagnosed after complete history consultation, physical examination, and routine laboratory examinations for 1 week. If the patient had fever in hospital and was suspected of bacteremia, and if the patient was immunocompromised, the criteria might be a little wider which was determined by the treating clinicians.

Immunocompromised refers to a person who has a significantly impaired immune system according to the National Institute for Health and Care Excellence (NICE) guideline. For instance, this may be because of prolonged corticosteroid use, tumor necrosis factor-alpha antagonists, antirejection therapy, immunosuppression-causing medication or comorbid states that affect the immune system, for example, HIV, chronic renal disease, many haematological and solid cancers, and diabetes [6].

### Laboratory diagnosis

The laboratory diagnosis of MTB BSI was based on the following: Peripheral blood cultures were collected and inoculated into a liquid culture method (Bact Alert MP, BioMerieux) between 2008 and 2010 and BD MGIT960 (BD) after 2010. Recovery of mycobacteria confirmed by acid-fast stain (Ziehl-Neelsen stain and Auramine-rhodamine stain) according to “Manual of Clinical Microbiology” [7]. And then Tuberculosis Antigen Colloidal Gold Diagnostic Kit (Kabelykit. Protocol is available at http://www.hgb.com.cn/cp1/show/id/44.html) and gene chip [8] are used to identify the type of Mycobacterium species according to the manufacturer’s instructions.

The direct smear on clinical specimens is done by Auramine-rhodamine stain and Ziehl-Neelsen stain at the same time following the standard in “Manual of Clinical Microbiology” [7].

Four milliliters of peripheral blood were collected from each patient and was performed within six hours after collection by laboratory personnel blinded to patients’ clinical data. In the T-SPOT.TB test, AIM-V (GIBCOTMAIM V Medium liquid, Invitrogen, US) was used as negative control, phytohemagglutinin (PHA) as positive control, and early-secreted antigenic target 6-kDa protein (ESAT-6) and culture filtrate protein 10 (CFP-10) as specific antigens, respectively. Peripheral blood mononuclear cells (PBMCs) were separated by Ficoil-Hypaque gradient centrifugation and obtained (2.5 × 10^5^per well) on a plate (Oxford Immunotec, Abingdon, UK) that was pre-coated with an antibody against interferon-γ. After incubation for 16 to 18 h at 37 °C in 5% carbon dioxide, wells were washed and developed with a conjugate used and an enzyme substrate against the antibody. The number of spot-forming cells (SFCs) representing an antigen-specific T cell that secreted interferon-γ were counted with an automated ELISPOT reader (AID-ispot, Strassberg, Germany). A positive result of T-SPOT.TB on PBMCs was defined as six or more SFCs in the target well and had twice the number of spots than the negative control well. In addition, the background number of spots in the negative control well should be less than ten SFCs [9].

### Statistical analysis

We used Kolmogorov-Smirnov test to check if the variables followed normal distribution. Measurement data of normal distribution was expressed by mean ± standard deviation (SD), and measurement data of non-normal distribution was expressed by median and interquartile range (IQR). Enumeration data was expressed by percentage. The Pearson’s Chi-square test was used to compare proportions between groups. Results with a 2-sided *P* value <0.05 were considered statistically significant. Statistical analysis was performed by SPSS 16.0 (SPSS Inc, Chicago, IL, USA).

## Results

We searched the electronic database in the clinical microbiology laboratory at Peking Union Medical College Hospital, and found 56 reports of positive Mycobacterium species from peripheral blood samples between January 2008 and May 2014. Then we excluded eight cases of non-Mycobacterium tuberculosis (NTM), 48 patients were MTB BSI.

### General conditions

Patients‘characteristics are summarized in Table 1. Among the 48 patients, 25 patients (52%) were males and the median age was 44 years (IQR 26 ~ 57). In addition to the MTB BSI, 26 patients (54%) had pulmonary tuberculosis, among these patients 13 (50%) had positive sputum smear for AFB, six (23%) had positive sputum culture for MTB (four patients had smear and culture positive), and 11 (42%) had radiological findings suggestive of pulmonary tuberculosis with negative smear and cultures from sputum. Other organ(s) involvement included the central nervous system (CNS) in seven (14%) patients, serositis including pleuritis, peritonitis, and pericarditis in five (10%), liver in five (10%), osteo-articular joints in four (8%), skin and soft tissue in three (6%), lymphatic system in three (6%), intestinal tract in two (4%), and spleen in two (4%). Most of the patients (26, 54%) were immunocompromised due to their underlying conditions and/or being on immunosuppressive therapy and only two patients (4%) were positive for HIV with a CD4 count of 25/mm^3^ and 60/mm^3^ each (Table 1).

**Table 1:** Demographics and underlying conditions of the 48 patients with MTB BSI

Characteristic	Value (*n* = 48)
Gender-n.(%)	
Male	25 (52)
Female	23 (48)
Age—Year (Median, IQR)	44 (26 ~ 57)
Subgroup-year,n.(%)	
18–65	41 (85)
66–79	6 (13)
80–99	1 (2)
Immune suppression-n.(%)	26 (54)
HIV-infection	2
On Steroids	17
SLE	7
SS	2
RA	2
DM	2
MCTD	1
Undifferentiated CTD	2
Myasthenia gravis	1
Diabetes mellitus	3
Malignant tumor	3
Renal transplantation	1

*HIV* Human immunodeficiency virus, *SLE* Systemic lupus erythematosus, *SS* Sjogren Syndrome, *RA* Rheumatoid arthritis, *DM* Dermatomyositis, *MCTD* Mixed connective tissue diseases, *CTD* Connective tissue diseases

### Clinical manifestations

The most common clinical manifestation was fever with a median time from fever to diagnosis of MTB BSI of 8 weeks (IQR 5 ~ 14). The median peak temperature was 40 °C, (IQR 39.5 ~ 40.1). Other clinical manifestations included cough with sputum production in 22 patients (46%), reported weight loss in 23 patients (48%), and night sweats in seven patients (15%). In addition, 12 patients (25%) had enlarged lymphatic nodes, 17 (35%) had hepatomegaly, and 27 (56%) had splenomegaly.

### Laboratory examinations

The laboratory findings are depicted in Table 2. Among the 41 patients who had a T-SPOT.TB assay done, 28 (68%) were positive with a median spots counts of 556 SFC/10^6^PBMC, (IQR 112 ~ 1704) (Table 3). The sensitivity of the T-SPOT.TB assay in patients with CD4 count > 200/mm^3^ was 100% compared to 57% in patients with CD4 counts < 200/mm^3^ (*P* = 0.018). When we looked at the absolute lymphocytes count (ALC), the sensitivity of the T-SPOT.TB assay was much higher in patients with ALC > 500/mm^3^ when compared to patients with ALC < 500/mm^3^ (85% vs. 52%, respectively, *P* = 0.025) (Table 4).

**Table 2:** Laboratory findings on 48 patients with MTB BSI

Characteristic	Value (*n* = 48) (n.%)
Leukocytopenia	24 (50)
Anemia	38 (79)
Low platelet count	28 (58)
High leukocyte count	17 (35)
Leukocytes, ×10^9^/L [median (IQR)]	4.58, (1.94 ~ 13.67)
Hemoglobin, g/L[mean ± SD]	88 ± 32
Platelets, ×10^9^/L[median (IQR)]	74, (25 ~ 213)
Lymphocytes, ×10^9^/L [median (IQR)]	0.35, (0.20 ~ 0.74)
Increased ESR	34/45(76)
Increased hsCRP	34/36 (94)
ESR, mm/h [median (IQR)]	69, (IQR 27 ~ 103)
hsCRP, mg/L [median (IQR)]	121.5, (IQR 56.4 ~ 176.5)
CD4+ count, /mm^3^, [median (IQR)] (*n* = 27)	115, (IQR 77-187)
CD8+ count, /mm^3^, [median (IQR)] (*n* = 27)	168, (IQR 78-246)
Time from MTB culture to a positive result, d[median (IQR)]	27, (IQR 21 ~ 33)

*ESR* Erythrocyte sedimentation rate, *hsCRP* Hypersensitive C reactive protein

Leukocytopenia: <4 × 10^9^/L; Anemia: Male < 120 g/L and female < 110 g/L; Low platelet count: <100 × 10^9^/L; High leukocyte count: >10 × 10^9^/L; Increased ESR: Male > 15 mm/h and female > 20 mm/h; Increased hsCRP: >3 mg/L

**Table 3:** Results of the T-SPOT.TB assay on 41 patients

T-SPOT.TB (SFC/10^6^PBMC)	MTB
Positive results	28/41 (68%)
Total	556 (IQR 112 ~ 1704)
ESAT-6	116 (IQR 34 ~ 242)
CFP-10	100 (IQR 40 ~ 722)
Negative results	13/41 (32%)

ESAT-6: 6 kDa, specific antigens encoded in the RD1 region; CFP-10: 10 kDa, specific antigens encoded in the RD1 region

**Table 4:** T-SPOT.TB results stratified by CD4 counts and absolute lymphocytes count

	T-SPOT.TB positive (n, %)	T-SPOT.TB negative (n, %)
CD4 < 100/mm^3^(*n* = 10)	4 (40)	6 (60)
CD4 100-200/mm^3^(*n* = 11)	8 (73)	3 (27)
CD4 > 200/mm^3^(*n* = 6)	6 (100)	0 (0)
*P* value	0.018	
ALC < 500/mm^3^(*n* = 21)	11 (52)	10 (48)
ALC > 500/mm^3^(*n* = 20)	17 (85)	3 (15)
*P* value	0.025	

*ALC* Absolute lymphocyte count

### Treatment and outcome

Data on therapy was available for 39 patients as the remainder nine patients were transferred to other facilities before any therapy was initiated. All the patients received at least three out of the four drugs of the standard regimen (Isoniazid 0.3 g/d, Rifampin 0.45 g/d, Pyrazinamide 0.75 g/d, and Ethambutol 0.75 g/d) and in combination with a quinolone in 27 patients (Moxifloxacin 0.4 g/d (*n* = 10), and Levofloxacin 0.5 g/d (*n* = 17)).

In total 25 patients were lost to follow-up (Nine patients were transferred to other facilities before any therapy was initiated and 16 were lost to follow-up prior to completing treatment). 14 (36%) recovered well without major complications, and one patient with tuberculous meningoencephalitis recovered but with subsequent seizures with a median duration of 12 months of treatment (range 6–30 months). The remaining eight patients (20.5%) died at a median of 2 days (range: 2 to 150 days) from diagnosis (seven died of TB, and one of gastric perforation).

Among the 26 immnocompromised patients, six patients (23%) died of TB, while among 22 immunocompetent patients, one patient (5%) died of TB (*P* = 0.07), they all had poor outcomes.

With a comparison of the 15 patients recovered with those eight patients died, median time from onset of symptoms to receiving treatment was 30 days (IQR 17-60) and 55 days (IQR 37-96), respectively (*P* = 0.023).

## Discussion

We report a large series of patients with MTB BSI. Our data showed that patients with MTB BSI usually have multiple organs involvement with varied serious complications. In addition, the time to diagnosis could be long and the outcome was generally poor. On the other hand, we found that sensitivity of T-SPOT.TB assay was high mainly in patients with CD4 count above 200/mm^3^.

Tuberculosis remains one of the most challenging communicable diseases in the world. In 2013, an estimated 9.0 million individuals developed TB and 1.5 million died. China alone accounted for 11% of the total cases worldwide [10]. Although pulmonary tuberculosis is the form given the most attention for its public health relevance, other forms of TB, including MTB BSI are also important, particularly when more people are becoming immunocompromised due to cancers, or other chronic diseases. Interestingly, in one study conducted in Malawi showed that MTB ranked second at 19% as etiology of bloodstream infections [4]. MTB BSI was more likely to occur in HIV-positive than in HIV-negative patients (13/173 vs. 0/65; *P* < 0.05) [4]. MTB BSI in patients with HIV infection or other immunosuppressive diseases resulted in death in most patients [2, 3, 11]. Whether early and prompt diagnosis and treatment would have impact on the overall prognosis should be determined in future studies.

Hematogenous, disseminated, or miliary tuberculosis has been used exchangeably in the literature describing in most instances a lymphohematogenous spread of MTB. Miliary tuberculosis accounts for about 1–2% of all cases and about 8% of all forms of extrapulmonary TB in immunocompetent individuals. These forms of infections are more frequently encountered in immunocompromised patients [1, 12, 13] as seen in our study where the majority of patients (54%) had an underlying immunosuppression. We hypothesize that initial exposure occurs through the respiratory route that may or may not result in pulmonary TB at later stages dependent most probably on the inoculum effect and the virulence of MTB in addition to the immune status of the host. Dissemination could occur through the blood and seed many organs, oxygen rich organs in particular, such as the brain, bones, lungs, and spine.

Not surprisingly, the most common organ involved in patients with MTB BSI was the lungs (54%). The radiological manifestations were atypical in early stages and included, either normal findings, or ground-glass opacities, and progressed to miliary nodular patterns at later stages. A retrospective study of 2016 cases with culture-proven TB [14] showed that miliary and pneumonic radiographic patterns were risk factors for fatal outcomes. In addition, previous study [1] supported that there is an association between the occurrence of miliary MTB and a massive lymphohaematogenous dissemination of the pathogen from a pulmonary or extrapulmonary source resulting in embolization of the vascular beds of various organs. Subsequently, cerebral, hepatic or splenic involvement with MTB was most probably preceded by the hematogenous spread of this pathogen. Whether early diagnosis of MTB BSI in high endemic countries, like China, may prevent seeding of MTB in different organs with prompt therapy should be determined in future studies.

We also evaluated the performance of the T-SPOT.TB assay among patients with MTB BSI. The overall sensitivity was 68%, lower than what is reported in the literature [6, 15]. Although this assay performed well in patients with underlying immunosuppressive conditions, the combination of disseminated TB and the immunosuppressive status of some of our patients with low CD4 counts, may have contributed to this low sensitivity for the diagnosis of active TB. Similar results was found in another study that the sensitivity of the TB ELISPOT assay decreased from 97 to 81% when patients were stratified according to absolute lymphocytes count (ALC) above 1000/ml or less than 500/ml, respectively (*P* = 0.007) [16].

As the only confirmatory test available, the turnaround time for culture is long, which makes the diagnosis challenging and delayed. Median culture time of MTB was 26.6 days in our study which was similar to other report [17]. On another hand, it is recommended more than one blood specimen to be collected to increase the diagnostic yield for cultures [17]. We may apply the new method of GeneXpert MTB/RIF in the future which can detect MTB in 2 h with a good sensitivity and specificity [18]. It allows rapid laboratory confirmation of MTB and that empiric therapy is warranted pending laboratory confirmation, especially when disseminated TB is likely.

There are some limitations in our study. This is a retrospective study from a single referral tertiary care hospital caring most for complicated and serious diseases in China. And some patients were lost to follow-up. So our findings may not be generalizable to other patients in other centers.

## Conclusions

In summary, MTB BSI is a serious complication even among non-immunosuppressed patients. Multiple organs involvement is common and is associated with poor outcomes. Rapid diagnosis and prompt therapy may favorably impact outcomes.

## Abbreviations

AFB: Acid-fast bacilli
ALC: Absolute lymphocytes count
BSI: Blood stream infection
CFP-10: Culture filtrate protein 10
CNS: Central nervous system
ESAT-6: Early secretory antigenic target 6
HIV: Human immunodeficiency virus
MTB: Mycobacterium tuberculosis
SFCs: Spot-forming cells
